# The Therapeutic Effect of SCFA-Mediated Regulation of the Intestinal Environment on Obesity

**DOI:** 10.3389/fnut.2022.886902

**Published:** 2022-05-17

**Authors:** Huimin You, Yue Tan, Dawei Yu, Shuting Qiu, Yan Bai, Jincan He, Hua Cao, Qishi Che, Jiao Guo, Zhengquan Su

**Affiliations:** ^1^Guangdong Engineering Research Center of Natural Products and New Drugs, Guangdong Provincial University Engineering Technology Research Center of Natural Products and Drugs, Guangdong Pharmaceutical University, Guangzhou, China; ^2^Key Laboratory of Glucolipid Metabolic Disorder, Guangdong TCM Key Laboratory for Metabolic Diseases, Guangdong Metabolic Disease Research Center of Integrated Chinese and Western Medicine, Ministry of Education of China, Guangdong Pharmaceutical University, Guangzhou, China; ^3^School of Public Health, Guangdong Pharmaceutical University, Guangzhou, China; ^4^School of Chemistry and Chemical Engineering, Guangdong Pharmaceutical University, Guangzhou, China; ^5^Guangzhou Rainhome Pharm & Tech Co., Ltd, Guangzhou, China

**Keywords:** SCFA, gut microbiota, intestinal environment, obesity, metabolism

## Abstract

Intestinal environment disorder is a potential pathological mechanism of obesity. There is increasing evidence that disorders in the homeostasis of the intestinal environment can affect various metabolic organs, such as fat and liver, and lead to metabolic diseases. However, there are few therapeutic approaches for obesity targeting the intestinal environment. In this review, on the one hand, we discuss how intestinal microbial metabolites SCFA regulate intestinal function to improve obesity and the possible mechanisms and pathways related to obesity-related pathological processes (depending on SCFA-related receptors such as GPCRs, MCT and SMCT, and through epigenetic processes). On the other hand, we discuss dietary management strategies to enrich SCFA-producing bacteria and target specific SCFA-producing bacteria and whether fecal bacteria transplantation therapy to restore the composition of the gut microbiota to regulate SCFA can help prevent or improve obesity. Finally, we believe that it will be of great significance to establish a working model of gut– SCFA– metabolic disease development in the future for the improvement this human health concern.

## Introduction

Obesity is among the largest health epidemics of the twenty first century, with a prevalence of ~13% in the world's adult population, and it is a major contributor to global diseases such as diabetes, cardiovascular disease and cancer and premature death (1, 2). Obesity is strongly associated with intestinal health, and the intestinal environment of obese patients is obviously disturbed (3, 4). On the one hand, because the intestine is continuously exposed to antigens from food, the microbiota and metabolites, intestinal inflammation is considered the origin of metabolic diseases. However, until recently, the relationship between intestinal inflammation and immune cell changes and obesity and insulin resistance has not been thoroughly studied (5–8). Increasing evidence shows that a high-fat diet (HFD) is related to low-grade intestinal inflammation. The intestinal immune system changes during HFD feeding. Therefore, inhibiting intestinal inflammation may be a treatment for obesity (6, 9, 10). In addition, intestinal hormones produced by intestinal epithelial cells (IECs), which are related to affect nutrient absorption rate, enteric environment composition and epithelial barrier integrity, are also thought to be associated with obesity. Regulating the release of endogenous glucagon-like peptide-1 (GLP-1) and other intestinal hormones is considered a promising strategy for obesity treatment, and may even mimic bariatric surgery (11, 12). In addition, intestinal function is closely related to the body's energy metabolism. IECs can absorb lipids and package them into chylomicrons for delivery to peripheral tissues. In performing this function, intestinal cells consume up to 20% of all incoming energy and therefore represent the primary cell type utilizing energy in these tissues (13). However, there are few in-depth therapeutic studies on obesity-induced changes in the intestinal epithelial barrier, microbiota, and innate and adaptive immune cells.

To date, there is growing evidence that the gut microbiota regulates the effects of diet and alters host metabolism and the incidence of metabolic disorders. The gut microbiota produces a wide range of metabolites that act as messengers between microorganisms and their hosts. Microorganisms produce these metabolites as intermediates in their constituent metabolic pathways to chemically modify specific dietary components through enzymatic reactions unrelated to normal metabolism. Most of these metabolites have beneficial effects on the host (14, 15). Short-chain fatty acids (SCFA) are produced by bacterial fermentation of non-digestible carbohydrates (NDCs) (16, 17). The main SCFA in the intestinal tract are acetate, propionate and butyrate, which account for more than 95% of all SCFA. They are usually present in the gut at a concentration ratio of 3:1:1 (18, 19). SCFA have long been proven to affect different cell processes and functions in the intestine, such as cell differentiation, cell apoptosis, colon motility and electrolyte absorption (20–22). Therefore, to take advantage of gut bacterial metabolites such as SCFA and improve the intestinal environment by adjusting metabolism via SCFA-based treatment of obesity and related metabolic diseases, this review focuses on two aspects: first, the use of SCFA to regulate intestinal homeostasis to treat obesity and second, treatment based on targeting the role of gut microbes in SCFA production as obesity-related treatment.

## SCFA Mediate the Interplay Between Intestinal Homeostasis and Obesity

**Figure 1:**
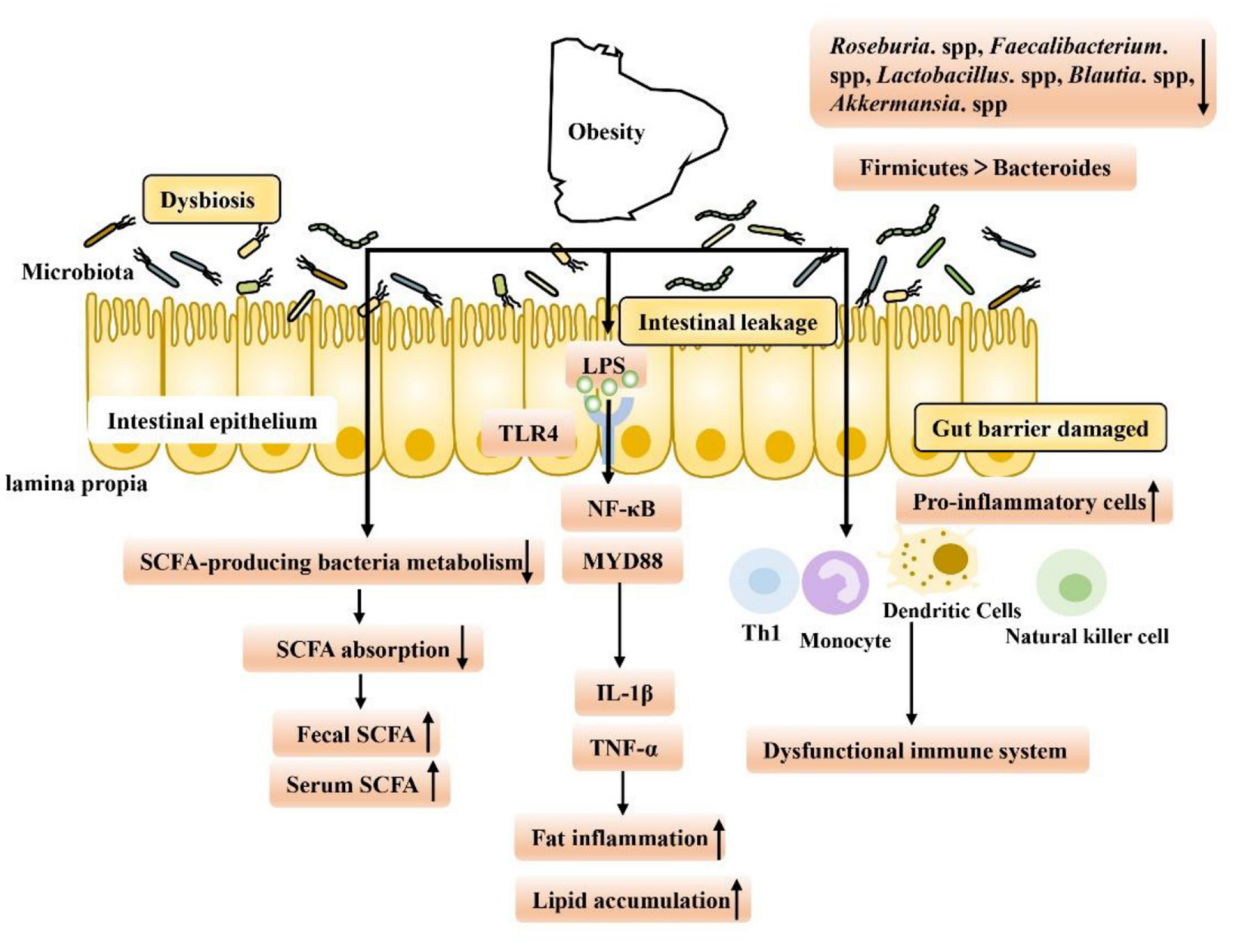
Gut microbiome disorders in obese patients are caused by intestinal environment disorders, which have the following characteristics: (1) Intestinal leakage and low levels of LPS in the blood may activate signal transduction in various TLR4-expressing cells, leading to the occurrence of fat inflammation and lipid accumulation. (2) Intestinal immune function decreased, and levels of proinflammatory Th1 cells, total macrophages, dendritic cells and NK cells increased, exacerbating inflammation. (3) The abundance of SCFA-producing bacteria decreased, leading to a decrease in the intestinal absorption capacity of SCFA and an increase in fecal SCFA concentration. Increased nutrient intake is associated with increased levels of gastrointestinal leakage, and through passive ingestion, increased serum SCFA concentration.

Evidence from nearly a decade of studies has shown that obesity is associated with gut dysfunction (Figure 1). First, intestinal dysfunction in obese patients is associated with intestinal dysbiosis, which has been found in HFD-fed animals. When dysbiosis occurs, the intestinal tract may gradually leak, allowing lipopolysaccharide (LPS) produced by gram-negative bacteria to enter the hepatoenteric circulation (3). Low levels of LPS in the blood may then activate signaling in various Toll-like receptor 4 (TLR4) cells, leading to systemic and local inflammation in HFD-fed mice. Overproduction of proinflammatory cytokines such as tumor necrosis factor-α (TNF-α), interleukin-1β (IL-1β), and interferon-γ (IFN-γ) in obese animals may be responsible for chronic inflammation and insulin resistance, in part through the nuclear factor kappa-B (NF-κB) pathway (7, 23–28). Second, intestinal immune function is also reduced in obese people. Researchers have found an increased number of white blood cells in the intestinal mucosal barrier, a shift to proinflammatory cell types (such as Th1 cells, total macrophages, dendritic cells, and natural killer (NK) cells) in obese people, and an increased incidence of colitis (6, 29, 30). Finally, it is well-known that an important feature of intestinal microbiota disorder is the decreased bacterial abundance of SCFA in the intestinal microbiota of obese patients, which leads to a decrease in SCFA levels and the occurrence of intestinal inflammation (31). SCFA, as key microbial metabolites that maintain the function of the intestinal epithelium, have been deeply studied regarding their ability to support intestinal barrier function (Table 1). On the one hand, SCFA can provide energy for the host epithelium. SCFA epithelial metabolism is a major determinant of hypoxia in the mucous membrane, and bacteria producing butyrate affect the oxygen consumption of epithelial cells and lead to the stabilization of hypoxia-inducible factor (HIF), a transcription factor that coordinates barrier protection and thus plays a key role in the maintenance of epithelial barrier function (53, 54). On the other hand, SCFA have been shown to increase the expression of the intestinal barrier-related genes tight junction protein 1 (TJP1), occludin (OCLN) and zonula occludens 1 (ZO-1) and inhibit the expression of Claudin 2 to repair the intestinal barrier (55–61). In obesity and related metabolic diseases, SCFA mainly inhibit histone deacetylases (HDACs) and activate SCFA-related receptors to maintain the intestinal environment. Based on this background, the following two aspects are focused on in the discussion below. First, we discuss SCFA-dependent receptor pathways in and out of cells and second, we discuss the SCFA receptor-independent pathways that repair intestinal barrier function in the course of obesity treatment, providing new ideas and potential targets for obesity treatment in the future.

**Table 1:**
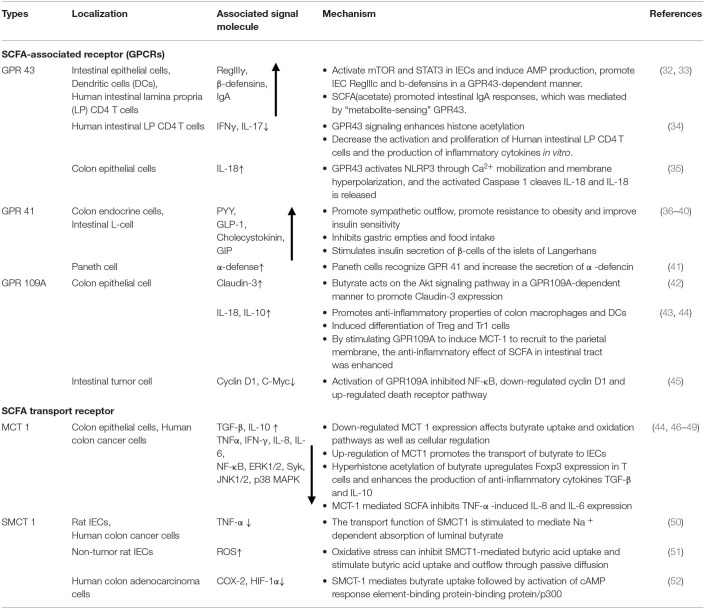
Location, signaling molecules and mechanisms of SCFA-associated receptor pathways in maintaining intestinal homeostasis.

### SCFA-Associated Receptor-Dependent Pathways

#### Extracellular SCFA Receptor Pathway and Obesity

SCFA-sensing G protein-coupled receptors (GPCRs), also known as free fatty acid receptors (FFARs), including GPR41 (FFAR3), GPR43 (FFAR2), and GPR109A, are mainly expressed by IECs, especially intestinal endocrine cells (31, 62, 63). They can be activated by SCFA and are highly expressed in the human intestinal mucosa, playing a key role in maintaining the homeostasis of intestinal barrier function. These receptors are present in the apical membrane of the colon epithelium, and luminal SCFA activate these receptors to function outside the cell without entering the cell (26, 64, 65). Activation of these receptors can stimulate the Gαs and Gβγ subunit signaling pathways to activate ERK, reduce the cellular level of cyclic adenosine monophosphate (cAMP) and increase intracellular Ca^2+^ levels, which act as a secondary messenger to initiate biological effects and activate various downstream signaling events, such as protein phosphorylation and changes in cellular behavior (66–68). The binding of GPCRs to β-inhibitory protein (β-ARR) also stimulates and promotes the activation of the Gαs and Gβγ subunits, thereby regulating *in vivo* physiological activities such as chemotaxis, apoptosis, proliferation, differentiation and gene expression (69–71).

**Figure 2:**
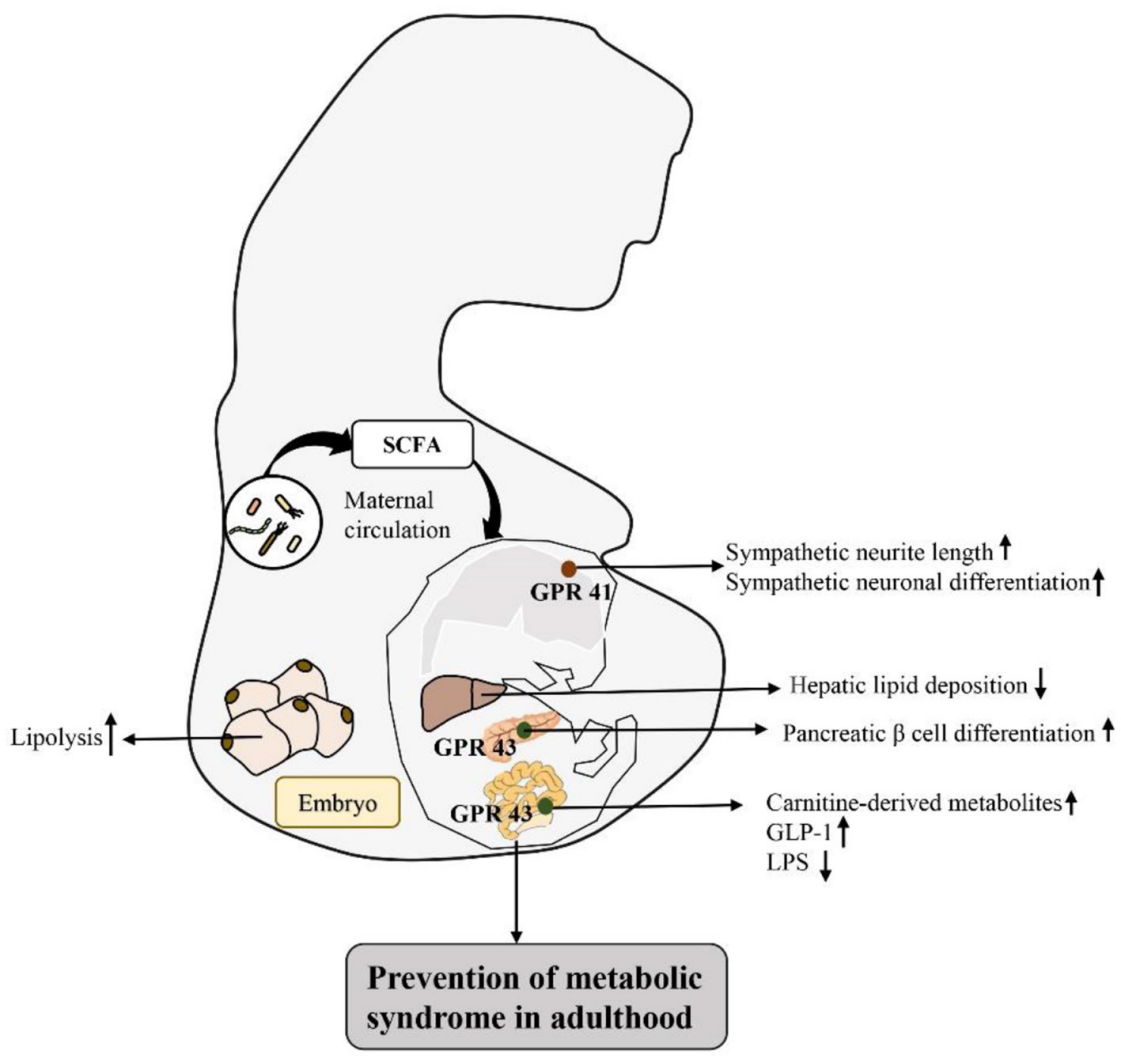
Activation of SCFA-associated receptors in early life intestinal response mechanisms to prevent metabolic diseases in adulthood: by regulating the intestinal flora matrix, increased maternal SCFA flows into the embryo to activate embryonic sympathetic nerve receptor GPR41 and intestinal epithelium and pancreas GPR43, promotes the development of the baby's nerve and bowel, improves embryo glucolipid metabolism, and improves the fetal malnutrition caused by maternal metabolic disorders, preventing metabolic diseases in adulthood.

**Figure 3:**
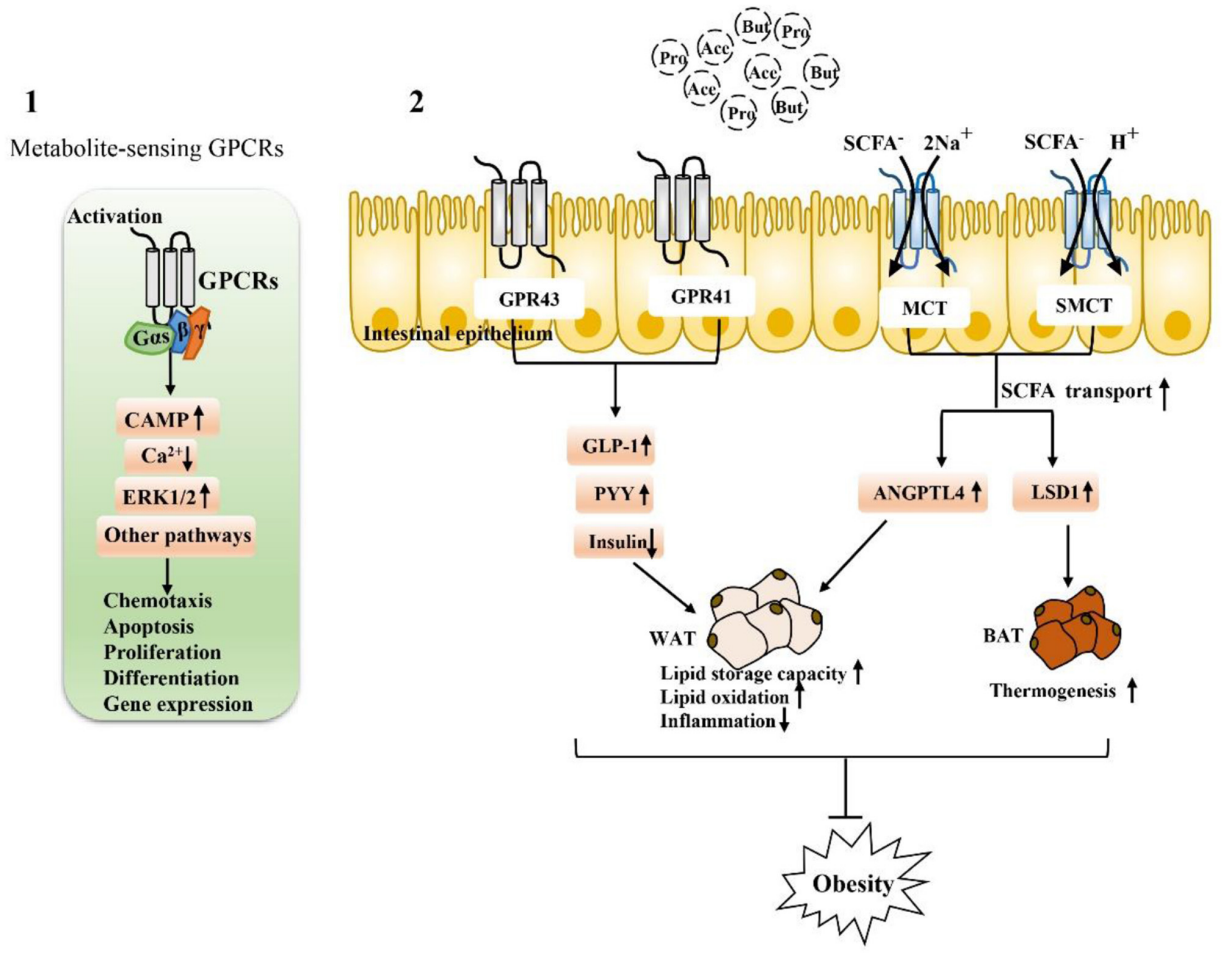
Activation depends on SCFA-associated receptors to maintain intestinal function and alleviate metabolic disorders: (1) Extracellular, SCFA-sensing G-protein-coupled receptors (GPCRs) are activated to recruit and bind downstream effector proteins (e.g., G proteins, β-arrestin, etc.), which then function *in vivo* through the cAMP signaling pathway, ERK signaling pathway 1/2, and calcium ion signaling pathway. (2) The activation of GPR43 and GPR41 receptors on intestinal endocrine cells is closely related to the regulation of metabolic disorders. After activation, GLP-1 can be stimulated, the secretion of PPY maintains glucose homeostasis, and the activation of SCFA transporters MCT and SMCT can promote the entry of SCFA into intestinal epithelial cells and regulate genes related to fat metabolism. Activation of these SCFA-dependent receptors is particularly important for fat storage, oxidation, inflammation, and thermogenesis.

Based on the response mechanism of SCFA receptor activation, many studies have proven that the activation/damage of SCFA receptors produces a significant response, especially in the intestinal tract, in early life (72) (Figure 2). HFD intake before and during pregnancy can change the composition of the maternal intestinal microbiota and ultimately lead to an increase in maternal circulating LPS levels. Reduction in maternal SCFA levels and inhibition of their receptor, GPR41, impairs the integrity of the intestinal barrier and may ultimately lead to changes in placental vasculogenesis and fetal intestinal development (73, 74). In a recent study, investigation of the SCFA-GPR41 and SCFA-GPR43 axes confirmed that offspring of germ-free maternal mice were more prone to obesity and glucose intolerance than offspring of non-specific pathogen-free female mice. SCFA produced by the gut microbiota of pregnant mice can enter the embryo through the blood. SCFA act on the sympathetic nerve receptor GPR41 and intestinal epithelial and pancreatic GPR43, promoting the expression of GLP-1 in nerve cells and intestinal endocrine cells and the differentiation of islet β cells. Other studies have shown that maternal supplementation with butyrate can increase the expression level of GPR43 in maternal adipose tissue, promote the decomposition of maternal white adipose tissue, reduce the liver lipid deposition of offspring, and play an important role in alleviating fetal malnutrition caused by metabolic disorders in maternal and fetal offspring. These results suggest that during pregnancy, intestinal microbiota and maternal microbiota SCFA promote a good environment for shaping the metabolic system development of embryos and energy homeostasis of offspring (75, 76). In addition, some researchers have attempted to establish that the SCFA-GPR43-GLP-1 pathway in the intestinal tract relieves hyperglycemia and found that activating this pathway had a good effect on the gut microbiota disturbance induced by tacrolimus and glucose metabolism disorder caused by ecological disorders; it also reversed the change in butyrate concentration in the cecum and increased the expression level of GPR43 in intestinal crypts. Moreover, this approach promoted GLP-1 secretion by L cells of the intestinal endocrine mucosa and restored fasting blood glucose (FBG), glycosylated hemoglobin (HbA1c) and oral glucose tolerance test (OGTT) values to normal levels (77). Therefore, SCFA, as an important regulator of body metabolism, play a key role in affecting adipose tissue metabolism, lipid oxidation capacity, β cell function and insulin secretion, suggesting that there is a mutual relationship among the microbiota, SCFA, and metabolism and that they may be potential targets for the treatment of obesity and related metabolic diseases (Figure 3). Therefore, we believe that targeting the gut microbiota to adjust SCFA levels to improve the intestinal environment and adjust glucose-regulating hormones in colonic L cells will in turn stimulate insulin secretion to improve glucose homeostasis, and if this approach is used particularly to intervene early in life, we can improve metabolism in adulthood. This strategy has great potential for treating metabolic diseases such as obesity.

#### Intracellular SCFA Transporter [MCT (SLC16A) and SMCT (SLC5A)] Expression

In addition to the extracellular functions described above, SCFA can also function intracellularly, which obviously requires their entry into colon epithelial cells. SCFA must pass through the epithelium to reach the serosa side to act on immune cells in the lamina propria (78, 79). Transport mechanisms are needed for intraluminal entry and transcellular transport; otherwise, SCFA cannot produce intracellular effects in colon epithelial cells and cannot act on mucosal immune cells. Moreover, at physiological colonic pH (5.6–6.6), the main form of SCFA is anionic and they cannot undergo simple diffusion. At the junction of the lumen, ionization of most SCFA is expected at physiological pH. In this regard, many studies have reported evidence for carrier-mediated absorption pathways for ionized SCFA (79, 80). Therefore, SCFA transporters expressed in the colon epithelium are also major determinants of the beneficial effects of SCFA on the host (78, 81–84). For example, H^+^-coupled transporters [monocarboxylate transporter (MCT)1/4] and Na^+^-coupled transporters [sodium-coupled monocarboxylate transporter (SMCT)1/2] transfer SCFA to colon epithelial cells and play specific roles in the absorption and utilization of SCFA in the intestine (Figure 3).

Recent studies have shown that the expression of SCFA transporters is effective in the treatment of obesity and related metabolic diseases. On the one hand, MCT1 is one of the main routes of butyrate transport in Caco-2 cells (85); when MCT1 expression is eliminated by siRNA, secretion of angiopoietin-like protein 4 (ANGPTL4) induced by *Clostridium butyricum* producing butyrate is greatly affected, thus affecting fat generation (86). In addition, it is worth noting that MCT1 has been proven to have certain effects on heat production and energy homeostasis. Inhibition of MCT1 can block the effect of butyrate in adipocytes. After inhibition of MCT1 expression, the effect of butyrate on LSD1 and UCP1 is completely blocked, and MCT1 has been proven to be a lactate transporter in adipocytes. It plays an important role in adipocyte function by mediating lactate flux (87). In addition, the Irving team first reported the relationship among SCFA, body mass index (BMI), and jejunum transporters in a cohort of morbidly obese patients undergoing bariatric surgery. The results showed that BMI was positively correlated with SMCT1 mRNA levels and that BMI was positively correlated with serum butyrate, valeric acid, and isocaproic acid concentrations. The increase in serum SCFA concentration may be due to increased uptake of SCFA in the colon, in part due to increased nutrient intake and the association of obesity with increased levels of gastrointestinal leakage, resulting in a complete bypass of SCFA transporters and increased passive uptake of SCFA. Interestingly, a new finding in this study is that although the jejunum was not a major site of SCFA uptake, increased serum SCFA concentrations were positively associated with the mRNA levels of the SCFA transporter SMCT1 in the jejunum (88). In summary, SCFA are essential for optimal colonic health. Transporters responsible for the entry and cross-cell transfer of these bacterial products into the intestinal epithelium are key determinants of maintaining intestinal function under physiological and disease conditions. By influencing the expression of SCFA transporters to regulate the intestinal tract, extraintestinal tissues and serum SCFA concentration can be used as a future therapeutic target to reduce the intestinal dysfunction caused by obesity.

### SCFA Receptor-Independent Pathway

**Figure 4:**
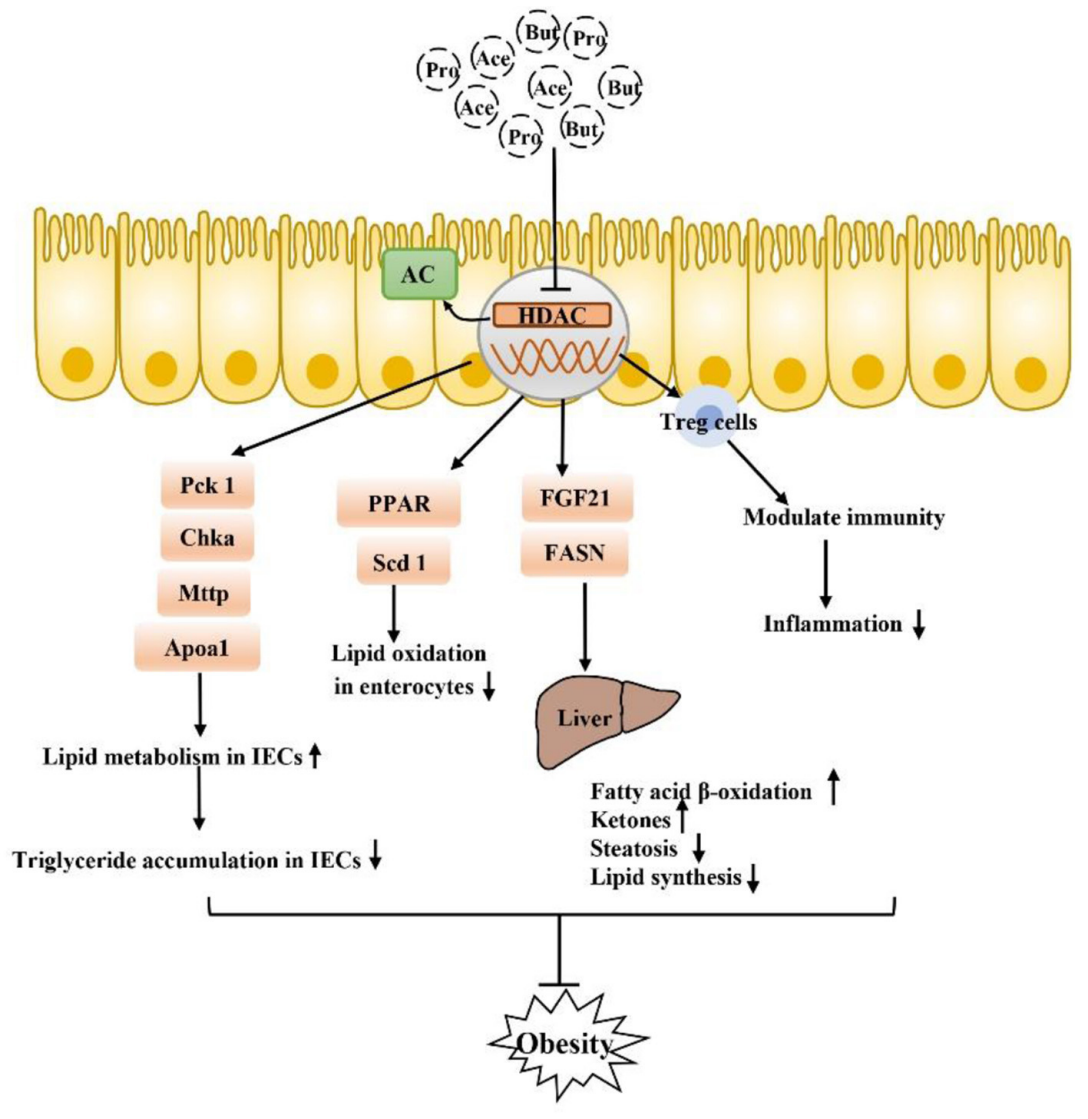
SCFA can also inhibit obesity by inhibiting the HDAC receptor-independent pathway: after inhibiting HDAC, SCFA play a core role in PPAR-regulated lipid oxidation in the intestinal epithelium and can regulate a variety of genes that respond to microflora regulation of metabolism to improve lipid accumulation in intestinal epithelial cells. Moreover, inhibition of HADA has been found to play a beneficial role in lipid metabolism in the liver, in addition to the regulation of Tregs in immune cells. Inhibition of inflammation is also closely related to the occurrence and development of obesity.

SCFA also function by inhibiting the receptor-independent pathway of histone deacetylases (HDACs) (Figure 4). HDACs are groups of deacetylases that remove acetyl groups from histone and non-histone complexes that regulate gene expression, and HDAC inhibitors have been shown to exhibit potent anti-inflammatory activity in inflammatory diseases (89–91). SCFA produced by intestinal microorganisms inhibit HDAC activity, which increases histone acetylation, relaxes chromatin structure and increases the accessibility of certain genes to transcription factors, thereby increasing their expression (92–95). SCFA-mediated HDAC inhibition is accelerated during intestinal barrier repair and plays a major role in the regulation of metabolic diseases. There is tremendous potential particularly for the use of the SCFA butyrate, which is a recognized HDAC inhibitor, in the treatment of obesity-related metabolic diseases (96–100). In IECs, a specific HDAC, HDAC3, can regulate histone acetylation in IECs, integrate microbiological signals to regulate intestinal homeostasis, and regulate lipid metabolism genes in a variety of tissues. HDAC3 expression in the small intestine in children is positively correlated with body weight, which was specifically observed with HDAC3 knockout in mouse IECs. The lack of HDAC3 changed the expression of genes (Chka, Mttp, Apoa1, and Pck1) that respond to the regulation of metabolism in IECs, increased the expression of β-oxidation-related genes in mitochondria and peroxisomes, and increased the oxidation rate of fatty acids. HDAC3 has also been found to inhibit genes involved in fatty acid oxidation in intestinal cells by inhibiting transcriptional targets in the peroxisome proliferator-activated receptor (PPAR) nuclear receptor family, which play a central role in coordinating PPAR regulation of lipid oxidation in the intestinal epithelium. In addition to artificially reversing the reduced weight loss in obese mice, fibroblast growth factor 21 (FGF21) gene expression in the liver and hepatocytes can be induced by inhibiting HDACs, thus stimulating the β-oxidation of long-chain fatty acids and the production of ketone bodies, which may be a treatment for obesity (13, 99, 101). Thus, SCFA (mainly butyrate) prevent diet-induced obesity by inhibiting HDAC3 activity in IECs, and attempts to regulate the butyrate-HDAC3 pathway may be used to prevent and ameliorate obesity-related diseases. Changes in the gut microbiota have been thought to affect intestinal inflammation and obesity. Antibiotic treatment can affect weight gain by eliminating microbes that produce HDAC inhibitors, which have anti-inflammatory effects by increasing regulatory T (Treg) cell counts, but a new study found that mice without HDAC6 did not show resistance to obesity but rather showed accelerated weight gain. HDAC6 deficiency may lead to enhanced activation of CD4+ and CD8+ T cells and imbalance of the gut microbiota; accordingly, the representation of the S24–7 family and Lactobacillus decreases, Bacteroides and Parabacteroides abundances increase, which promotes obesity (102). In summary, the microbial communities of obese individuals and the inhibition of HDACs exhibit a strong relationship. In conclusion, there is a strong correlation between the microbiome of obese people and the inhibition of HDAC, and the relationship between obesity and HADC needs to be further explored. SCFA, especially butyrate, as HDAC inhibitors, play a beneficial role in sustained low-grade inflammation in obesity and especially in balancing pro-inflammatory and anti-inflammatory immune responses, which has great research significance for the treatment of obesity.

## SCFA-Based Microbial Therapy

**Figure 5:**
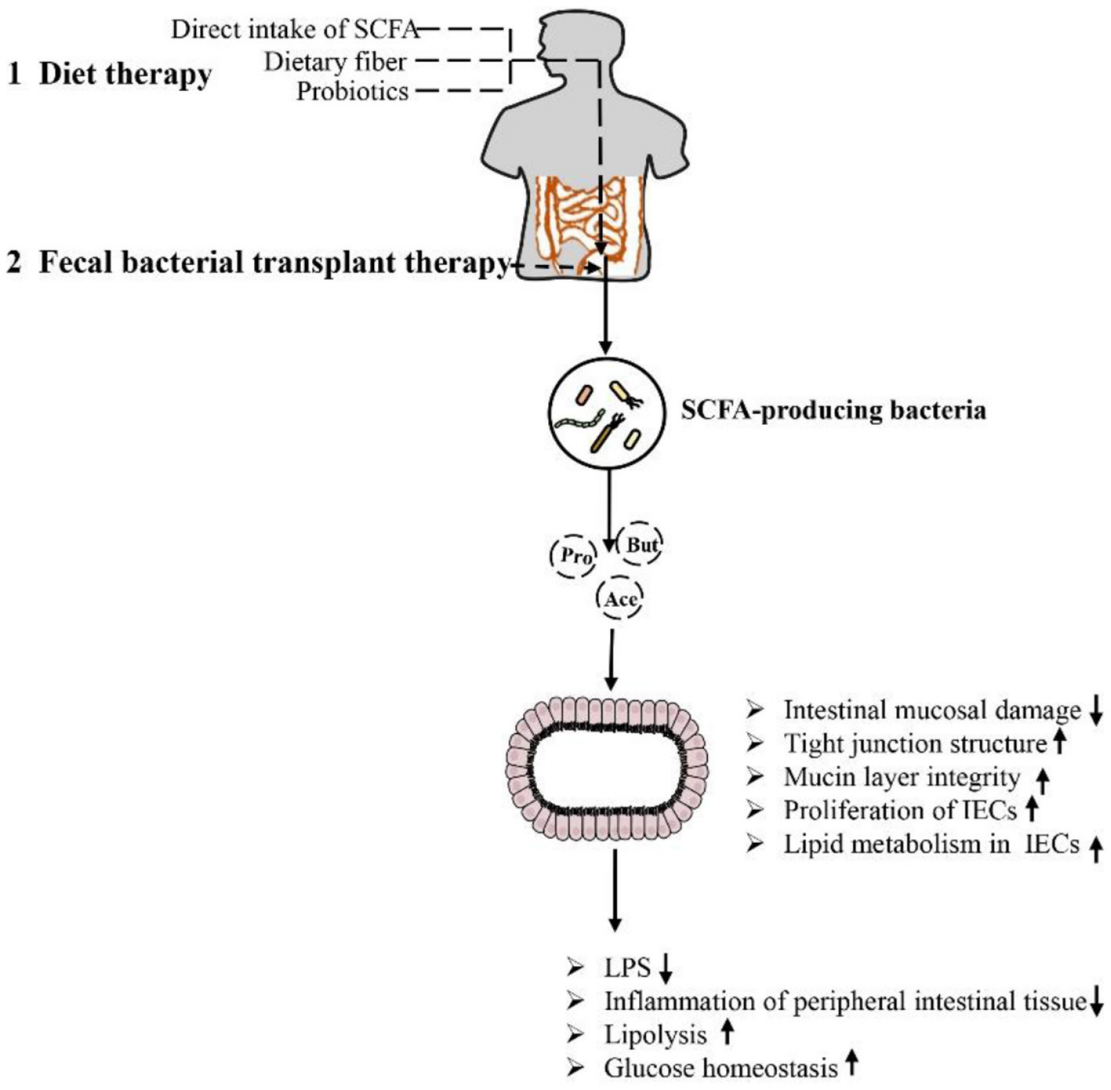
Methods and approaches to improve obesity-related metabolic diseases by regulating the intestinal environment through SCFA: Dietary therapy (direct supplementation of SCFA, dietary fiber and prebiotics) and microbiological therapy (fecal bacteria transplantation) concentrate/target SCFA-producing bacteria, increase SCFA production, repair intestinal barrier damage, reduce inflammation in intestinal and periintestinal tissues, enhance lipolysis, and improve glucose homeostasis. This approach results in inhibition of the occurrence of obesity and related metabolic diseases.

As mentioned above, SCFA have a strong relationship with intestinal microbial metabolites in the treatment of obesity and the repair of intestinal function. The role of SCFA is closely related to intestinal microbes. The microbiota of the human gut affects the metabolism of the host in various ways and may play a role in the development of obesity. Some species of microbes can play specific roles (26, 103). For example, at the genus level, *Blautia* is the only bacterium for which its abundance is negatively correlated with visceral fat area, which may be related to its ability to produce SCFA to regulate GPR41 and GPR43, promote fat decomposition and reduce fat accumulation (104, 105). In addition, it is recognized that the Firmicutes/Bacteroidetes ratio is increased in obese animals and humans, but acetate and propionate are mainly produced by Bacteroidetes, while butyrate is mainly produced by Firmicutes (18, 106, 107). The increased Firmicutes/Bacteroidetes ratio in obese individuals implies higher butyrate and lower propionate and acetate production in these subjects, a finding partially contradicted by the respective anti-obesogenic and obesogenic effects of these SCFA. According to the literature, there may be fewer SCFA-producing bacteria in obese individuals, and these bacteria are gradually replaced by other bacteria belonging to the same phylum, resulting in reduced production of SCFA in the colon (108). For example, the abundance of *Faecalibacterium prausnitzii* (Firmicutes), which is thought to be associated with obesity, has been shown to be significantly reduced in obese children (109, 110). Therefore, it is necessary to maintain the abundance and diversity of SCFA-producing bacteria, and attempts to regulate the species of SCFA-producing bacteria may have a beneficial effect on the treatment of obesity. In addition, it is worth mentioning that in the feces, the SCFA (butyrate, propionate, and isovaleric acid) concentration gradually increased with the increase in body weight and the increase in the level of microbial metabolism associated with obesity, but by adding SCFA, the host intake of SCFA triggered by the increase in the gut microbiota can cause the fecal SCFA concentration to be further reduced (26). In summary, certain drugs and microbe-oriented therapies can be used to help obese adults lose weight, triggering the metabolism of gut microbes in the body. In particular, some bacteria can increase SCFA production, thereby increasing the integrity of IECs, which can become one of the means for the treatment of obesity and related metabolic diseases (Figure 5).

### Diet Therapy

**Figure 6:**
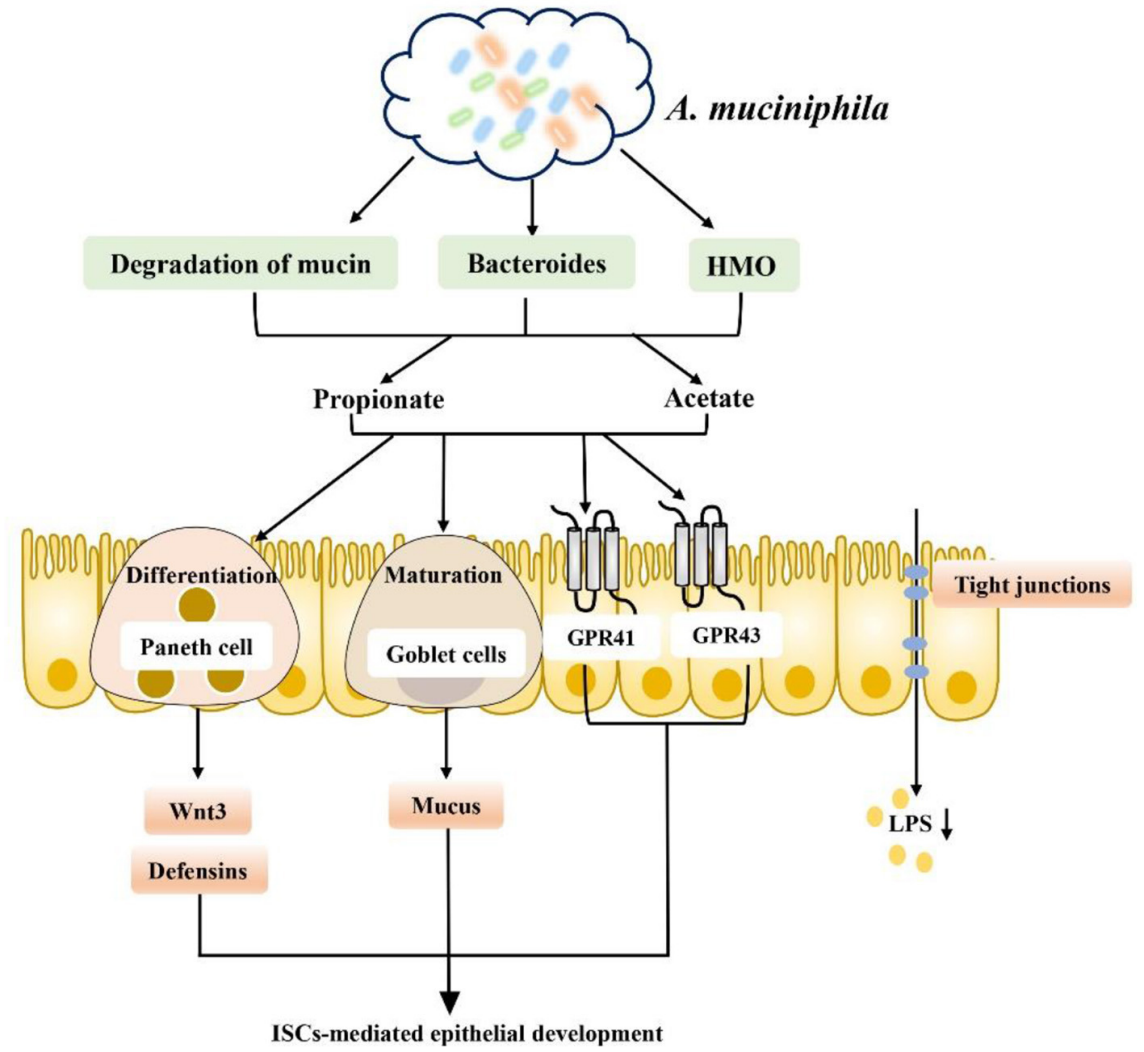
*A. muciniphila* promotes SCFA metabolism and improves the intestinal environment to improve metabolic abnormalities: (1) *A. muciniphila* can increase the production of SCFA (such as propionate and acetate) by degrading mucin, increasing the abundance of Bacteroidetes and degrading HMO. (2) Promoting intestinal epithelial cell proliferation by supporting mucus-secreting goblet cell differentiation, promoting the maturation of Wnt3- and defensin-secreting Paneth cells in a GPR43/41-dependent manner. (3) Promoting the secretion of GLP-1 and PYY in a GPR43/41-dependent manner. (4) Inducing Fiaf expression and inhibiting LDL production. (5) Inhibiting LPS production and inflammation.

It is well-known that dietary and microbiota interactions may activate bacterial metabolic pathways and functional metabolites associated with fat deposition in humans (111–114). Recent studies indicate that SCFA produced by carbohydrate fermentation are necessary for maintaining human health from an ecological perspective and can be considered an ecosystem service provided by the gut microbiota to human hosts. Restoring or enhancing lost or missing functions by re-establishing functional active ecological populations as ecosystem service providers (ESPs) is key to building a healthier microbiome, which helps mitigate disease. Targeted promotion of SCFA producers as ESPs through personalized nutrition may provide a new ecological approach for controlling gut microbiota to manage dysbiosis-related diseases (115). Therefore, dietary interventions to regulate the gut microbiota and increase the level of SCFA in the intestinal tract to repair the intestinal barrier, reduce inflammation caused by obesity and inhibit the occurrence of obesity and related diseases have become current hot spots. For example, dietary supplementation with sodium butyrate can inhibit histone deacetylation and activate signal transducer and activator of transcription 3 (STAT3), and induction of α-defensin expression in Paneth cells reduces intestinal barrier dysfunction in obese mice induced by HFD and corrects the intestinal microbiota imbalance in mice induced by HFD. In addition, butyrate increased the abundances of beneficial bacteria such as *Christensenellaceae, Blautia* and *Lactobacillus* that produce butyrate in a seemingly virtuous cycle. Thus, butyrate repaired intestinal mucosal damage, improved tight junction structure, reduced intestinal endotoxin entry into the liver, and alleviated liver inflammation and lipid accumulation caused by HFD consumption (116, 117). In addition, SCFA-producing bacteria such as *Ruminococcaceae, Muribaculaceae* and *Akkermansia* can also be enriched directly through diet to increase the level of SCFA to increase the expression levels of intestinal tight junction mRNA and protein and reduce intestinal permeability, improving intestinal barrier function in obese mice (118). Finding specific SCFA-producing bacteria and using diet or drugs to target certain bacteria is also a new therapeutic approach. For example, studies have found that strains of *Anaerostipes* and *Dysosmobacter welbionis* can metabolize inositol into propionate and butyrate to regulate the intestinal homeostasis of the body, which is associated with higher heat production of the body and may be beneficial to patients with obesity and diabetes (119–121). In a randomized clinical study of a specifically designed isoenergy diet, Zhao et al. used a bacteria-level, microbiome association approach to characterize the dynamics of the gut microbiome and its effect on glycemic homeostasis in patients with type II diabetes and identified a group of bacterial strains that produce acetate and butyrate. These strains are selectively promoted by increasing the availability of a variety of fermentable carbohydrates in the form of dietary fiber. These SCFA producers may be key players in maintaining a mutually beneficial relationship between the gut microbiome and the human host, playing a key role in maintaining a healthy gut environment (115). Additionally, a new strain of *Akkermansia muciniphila* isolated from healthy human feces plays a critical role in maintaining mucin layer integrity, thereby reducing proinflammatory lipopolysaccharide translocation and controlling fat storage, adipose tissue metabolism and glucose homeostasis (122–124) (Figure 6). Researchers also established a cAMP-responsive binding protein H (CREBH)-deficient mouse model to demonstrate that the use of *A. muciniphila* administration ameliorated chronic hypertriglyceridemia caused by deletion of the CREBH gene in mice. This effect is mediated by upregulation of hepatic LDL receptor expression to facilitate clearance of triglyceride-rich lipoprotein residues from circulation. Moreover, such treatment improved glucose intolerance and reduced hepatic inflammatory stress caused by CREBH depletion (125). The role of *A. muciniphila* is closely related to SCFA. *A. muciniphila* has been proven to use mucin as an energy source and transform it into SCFA (acetate and propionate); on the other hand, SCFA production was increased by altering the abundance of Bacteroidetes, which produce acetate and propionate. Moreover, SCFA play a key role in supporting the differentiation of mucus-secreting goblet cells, promoting the maturation of Wnt3- and defensin-secreting Paneth cells, and promoting the proliferation of IECs in a GPR43/41-dependent manner (117, 123). In addition, *A. muciniphila* can degrade human milk oligosaccharides (HMO) to produce acetate and propionate to maintain a healthy mucosal layer and metabolic state under *in vitro* culture conditions containing breast milk (126). Based on its regulation of intestinal homeostasis, *A. muciniphila* has also been proven to have potential effects for the treatment of lipid metabolism disorders. Some researchers have observed that *A. muciniphila* has potential effects on lipid metabolism in host IECs. Several key transcription factors and genes involved in fatty acid, cholesterol and bile acid metabolism have been identified. For example, genes encoding liver x receptor (Lxr), carnitine palmitoyltransferase 1 (Cpt1), and hydroxymethylglutarate coenzyme A (HMG-CoA) synthase are all affected by *A. muciniphila*. In addition, Fiaf has been found to be related to obesity and can regulate lipid metabolism in different tissues, while *A. muciniphila* and its related SCFA metabolites can induce the expression of Fiaf, inhibit the generation of low-density lipoprotein (LDL), and maintain lipid homeostasis and metabolism in intestinal and peripheral tissues (127). The association between the gut microbiota and metabolic disease can be altered by diet. For example, metformin, commonly used in patients with type II diabetes, has recently been shown to alter the composition of the gut microbiota by enriching *A. muciniphila*, which degrades mucin, as well as several SCFA-producing microbiota constituents. Metformin's effect on the gut microbiota alters gut metabolomics, increasing the ability to produce butyric and propionic acids, with beneficial effects on glucose metabolism (128). In conclusion, intestinal barrier dysfunction is one of the key pathogenic factors of obesity, and SCFA generated by intestinal microbial metabolism play an obvious role in reducing intestinal leakage and inflammation of peripheral intestinal tissues, such as liver and adipose tissues, caused by obesity. Based on the level of intestinal microbial control of SCFA production, prevention of obesity-induced systemic inflammation may be a potential treatment for obesity and its related metabolic diseases in the future by using dietary interventions to enrich beneficial SCFA-producing bacteria or by directly targeting certain SCFA-producing bacteria to correct obesity-induced intestinal microbiome dysregulation.

### Fecal Bacterial Transplant Therapy

The composition of gut microbes can be changed by diet and prebiotics or probiotics, but a new and unexplored method altering the gastrointestinal microbiota called fecal microbiota transplantation (FMT) has become a hot research topic (129–131). In theory, FMT increases the diversity of the gut microbiota, alters microbial metabolism, limits intestinal permeability and reduces local and systemic inflammation. FMT has also been shown to be durable, to be mostly safe, and to have relatively few side effects (132–136). Recently, the treatment of obesity by FMT has attracted the attention of many researchers and is considered a potential treatment option for obese patients in the future (137–140). In a previously published study, Ridaura et al. (131) transplanted fecal flora from obese or lean identical twins into germ-free mice. The mice transplanted with the lean-associated microflora were reared with the mice transplanted with the corresponding obese-associated microflora, which inhibited weight gain and obesity-related metabolic phenotypes in the mice transplanted with the obese-associated microflora. The inhibition of obesity development was found to be associated with specific members of Bacteroidetes in the lean-associated microflora. Clinically, researchers have attempted to evaluate the efficacy of oral coated FMT plus supplementary fiber supplementation in a representative population with severe obesity and metabolic syndrome in North America and found that oral FMT can alter the microbial ecology of the recipient, thereby improving insulin sensitivity. Dietary fiber supplementation can enhance or maintain these effects, demonstrating that FMT can be an effective microbiological therapy (141). In addition, a recent preliminary clinical trial reported for the first time the short-term efficacy and safety of FMT in obese patients without metabolic disorders. Although FMT effectively and continuously changed the composition of the gut microbiota and bile acid metabolism of the subjects, making them more similar to those of lean donors, the BMI and SCFA and GLP-1 levels did not change significantly in the short term. However, during the experiment, it was observed that the butyrate-producing bacterium *F. prausnitzii* was implanted, and the gene level of butyrate-producing bacteria increased. Since it was not found in the obese microbiota, the researchers hypothesized that butyrate may be rapidly metabolized by colon cells, leaving no measurable content behind (142). In addition, it is worth mentioning that studies have found that although FMT can change the gut microbiota of obese subjects, it produces no significant improvement in body weight or most metabolic indicators. It seems unlikely that the changes in the composition of the microbiota induced by FMT alone can be sufficient to treat or prevent metabolic disorders in humans. It has been suggested that in the future, whether preselected or specifically designed microbial compositions of donors or recipients can optimize changes in beneficial microbiota constituents should be explored (143). This information indicates the inconsistent response rates and occasional adverse events of fecal bacterial transplantation, which illustrate the complexity of the treatment regimen. Attempts have also been made to find potential alternatives by using specific bacterial lysates (dissolved products of bacteria), specific components of bacteria or inactivated bacteria to treat diet-induced obesity in mice fed lysates of *Methylococcus capsulatus Bath*. Increased SCFA levels indirectly promoted Treg cell polarization after treatment, significantly enriched the abundance of Parabacteroides, increased FoxP3+RORγT+IL-17+ Treg cell levels, enhanced intestinal barrier function, and significantly improved diet-induced obesity (144). All these results suggest that FMT is effective in the treatment of obesity, but it is still necessary to determine the effect of the microbiota and its metabolites, such as SCFA, to develop more effective microbiota-based treatments for obesity in the future.

## Conclusion and Future Perspective

Obesity is a major global health problem, and its incidence is increasing each year. It is a chronic disease that affects various physiological systems, among which the intestinal tract has become the main target of study. In recent years, increasing evidence has shown that obesity is related to intestinal health. Studies have shown that the characteristics of the gut environment in obese individuals are related to the following aspects: intestinal immune responses promoting intestinal inflammation, reduction in intestinal microbial diversity and changes in intestinal microbial composition, damage to the integrity of the intestinal epithelial barrier, bacterial translocation, and increased LPS levels, decreased levels of intestinal hormones released by intestinal endocrine cells (GLP-1, GIP), and abnormalities in intestinal fat absorption and metabolism. These findings suggest that intestinal intervention is a promising target for the treatment of obesity and related metabolic diseases.

In the past decade, biological studies combining animal models and human studies have shown that gut microbes produce a large number of metabolites that regulate host responses and play an important role in human health. As one of the important metabolites of intestinal microorganisms, SCFA have a variety of beneficial effects on host energy metabolism regulation, especially in obesity and related metabolic diseases. As a bridge between obesity and intestinal homeostasis, SCFA are closely related to the pathogenesis of obesity and related metabolic diseases. SCFA are thought to be ligands that activate cellular signaling cascades. Specific cell signaling receptors, such as GPCRs, MCT and SMCT, and the epigenetic process, and, namely, the inhibition of HDACs, regulates the intestinal environment in obesity. Based on this knowledge, the first part of this review summarized the dependent pathways through which SCFA activate SCFA-associated receptors: (1) The establishment of the SCFA-GPR43/41-GLP-1 axis induces the release of endogenous GLP-1 and other intestinal hormones by intestinal epithelial cells (Colon L cells) to stimulate insulin secretion to improve glucose homeostasis, especially in the early stages of life. (2) By activating SCFA transporters (MCT and SMCT), which are responsible for the entry and transcellular transfer of SCFA into the intestinal epithelium, these receptors are closely associated with higher concentrations of SCFA in serum and feces *in vivo*, as well as intestinal permeability, markers of metabolic disorders, obesity and hypertension. Activation of SCFA transporter receptors can promote the transport of SCFA, increase the absorption and utilization of SCFA in the intestinal tract, regulate the concentration of SCFA in intestinal and extraintestinal tissues, reduce tissue inflammation caused by obesity, and regulate energy metabolism. (3) The SCFA non-receptor-dependent pathway inhibits the HDAC receptor-independent pathway. Interestingly, HDAC3, a specific HDAC, plays a significant role in integrating microbial signals to regulate intestinal homeostasis and regulate the expression of genes involved in lipid metabolism in a variety of tissues, especially through inhibiting transcription of the PPAR nuclear receptor family and inhibiting lipid oxidation. In addition, SCFA play a role in regulating gut microbes. The use of microbiome regulation has become an attractive strategy reported in several clinical studies. In this regard, the second part of this review summarized the use of microbial-oriented therapies to trigger the metabolism of intestinal microorganisms in the body, especially the enrichment of some SCFA-producing bacteria or the targeting of specific SCFA-producing bacteria, such as the representative *A. muciniphila* strain, to improve the intestinal environment to help obese adults lose weight. This is mainly done using the following two methods for enriching/targeting SCFA-producing bacteria to intervene in the occurrence and development of obesity. The first is the most common strategy: dietary management. SCFA-producing bacteria can be enriched/targeted by the use of certain dietary treatments or certain drugs to promote the production and transformation of SCFA by intestinal microorganisms. The second is a microbe-based therapy—FMT therapy—continuous FMT treatment improved the metabolic disorder of obese people, and could produce the effect mainly due to its effects on the metabolism of the gut microbes, and especially through the increase in microbial SCFA gene expression levels. However, it is worth noting that the microbiome is a complex ecosystem, and whether SCFA play a role directly or by regulating gut microbes remains to be explored. However, the clinical therapeutic effects of dietary management, drug therapy and fecal bacteria transplantation are still controversial and complicated, and there are inconsistent data on response rates and adverse events. Therefore, the physiological and pathophysiological functions of SCFA should be fully investigated, and we think in the future, further study of SCFA in obesity and related metabolic diseases and the mechanisms through which they affect the intestinal environment is required, and how to better apply the regulation of SCFA produced by gut microbes to treat diseases await exploration.

## Author Contributions

HY participated in the production, creation, and drawing of the manuscript. JG and QC participated in the revision, conception, and drafting of the manuscript. YT, DY, SQ, YB, JH, HC, and QC participated in the review and revision of the manuscript. All authors contributed to the article and approved the submitted version.

## Funding

This work was financially supported by the Science and Technology Program of Guangzhou, China (NO.202103000089), the Guangdong Demonstration Base for Joint Cultivation of Postgraduates (2019), the Science Foundation for Distinguished Young Scholars of Guangdong (2020B1515020026), and the National Natural Science Foundation of China (21804025).

## Conflict of Interest

QC was employed by Guangzhou Rainhome Pharm & Tech Co., Ltd. The remaining authors declare that the research was conducted in the absence of any commercial or financial relationships that could be construed as a potential conflict of interest.

## Publisher's Note

All claims expressed in this article are solely those of the authors and do not necessarily represent those of their affiliated organizations, or those of the publisher, the editors and the reviewers. Any product that may be evaluated in this article, or claim that may be made by its manufacturer, is not guaranteed or endorsed by the publisher.
